# The development and application of a two-step surveillance process for Healthy China Initiative based on wide coverage interagency data

**DOI:** 10.1186/s41256-023-00326-x

**Published:** 2023-09-22

**Authors:** Lin Liu, Xiaomeng Lan, Yili Yang, Yuying Luo, Xueli Zhang, Xiuli Wang, Jay Pan

**Affiliations:** 1 HEOA Group, West China School of Public Health, Sichuan University, No. 17 People’s South Road, Chengdu, 610041 China; 2 Institute for Healthy Cities and West China Research Center for Rural Health Development, Sichuan University, Chengdu, 610041 China; 3 Sichuan Health Information Center, Chengdu, 610041 China; 4 School of Public Administration, Sichuan University, Chengdu, 610041 China

**Keywords:** Health strategy, Healthy China Initiative, Surveillance process, Indicator system

## Abstract

**Background:**

*Healthy China* is a nationwide health strategy aiming at improving health from diverse dimensions, and strengthening high-quality assessment is essential for its stimulation. However, there is limited evidence in the surveillance of the actual performance of the initiative at regional levels. This study innovatively proposes a two-step surveillance process which comprehensively monitors *Healthy China Initiative* based on regional realities, thus provides guidance for policymaking.

**Methods:**

A flexible indicator system was firstly developed basing on Delphi survey and focus group discussions. And then the Analysis Hierarchical Process and the TOPSIS method were used to determine the weights of indicators and calculate comprehensive indexes as the surveillance outcomes. A pilot study was conducted in a typical area in China to verify the applicability of the process.

**Results:**

Following the surveillance process and basing on the implementation of *Healthy China Initiative* in the target region, an indicator system comprised of 5 domains and 23 indicators with weights was first developed specifically for the pilot area. Then 1848 interagency data of the study area were collected from 8 provincial institutions/departments to calculate the indexes and ranks of the five domains which were health level, healthy living, disease prevention and control, health service, and healthy environment. The outcomes showed that *Healthy China Initiative* in the pilot area had been constantly improved since the strategy proposed, while there were still issues to be tackled such as the deficient monitoring mechanisms and unevenly development progress.

**Conclusions:**

This study proposed a pragmatic surveillance process with indicators which could be tailored for specific context of target regions and produce meaningful surveillance outcomes to inform decision-making for policymakers, and also provided a theoretical foundation as well as empirical evidence for further health strategies and plannings assessment studies.

## Introduction

Health is an inevitable requirement for promoting human and social development, and health strategy is an advanced product for promoting health by integrating factors from multiple dimensions into account. The transition to the global agenda of Sustainable Development Goals brings new opportunities for countries to move forward toward achieving progress for better health, well-being, and universal health coverage [1]. In 1978, WHO and the United Nations International Children's Emergency Fund (UNICEF) held the International Conference on Primary Health Care and issued Almaty Declaration, which clearly pointed out the health strategy with a goal of “Health Care for All by 2000” [2]. In 2012, the UN General Assembly proposed the health strategy of Universal Health Coverage (UHC) claiming that everyone should have access to quality and affordable health care [3]. Both “Health Care for All” and UHC emphasized the importance of medical care for health, while the government of developed countries have practiced health strategies considering more health-related dimensions, such as *EU Health Programme*, *Healthy Citizen 2020* of US, *Healthy Japan 2035* [4–7]. These health strategies from developed countries have considered the impact of both anthropogenic environment and natural environment. To better promote these health strategies and thus improve people's wellbeing and health, the governments usually show more focus on the designing of appraisal dimensions and indicators tailored for local contexts and emphasize the necessity of routine surveillance and timely assessment. Experience from these developed countries would provide valuable guidance for the development of health strategies in China [8–11].

At the National Conference on Health and Wellness in 2016, president Xi Jinping has addressed the necessity of “holding people's health as a strategic priority” and deployed plans for *Healthy China*, which is the uppermost health strategy in China. Soon afterwards the State Council issued the *Healthy China 2030* Planning Outline [12], which described the specific goals and tasks for promoting people's health until 2030. In 2019, the document of Opinions on Implementing *Healthy China Initiative* [13] was proposed. This document further clarified the overall goals of the *Healthy China Initiative* and proposed 15 *Special Campaigns* to intervene in health influencing factors, control major diseases and improve health service system so that residents would have their health level significantly improved. Following the national deployment, most provinces have taken proactive measures to implement *Healthy China Initiative* based on local realities to take their measures to ensure people's health [14, 15].

The promotion of *Healthy China Initiative* at both national and regional levels requires effective monitoring and evaluation studies [16]. The importance of organization, monitoring, and regular assessment throughout the *Healthy China Initiative* was already emphasized by the State Council in 2019 [17]. Accurate and timely assessment would ensure the correct direction of *Healthy China Initiative*, and policymakers also need evidence-based information and assessment tools to facilitate efficient interventions to achieve timely appraisal and amendments [18]. The health systems of the low- and middle-income countries (LMICs), in particular, face the challenges of both infectious and parasitic diseases and chronic non-communicable diseases, posing further pressure on the relatively meager health system resources available. Knowledge of the stage of health transition of a country is necessary for priority setting and evaluation of health programs [1]. Considering the multi-dimensions included in the *Healthy China Initiative*, a well-designed indicator system is essential. It could serve as a potent monitoring tool in which an organic synthesis accountably reflects the characteristics and interconnections of the objects and could exactly describe the effects of the activities, facilitate accurate calibration, and proffer scientific decision-making suggestions [5, 10, 19]. Meanwhile, a metric comprehensive index is needed to show and compare the results of the surveillance in a simple and intuitive way.

While mature evaluation methodology has been proposed earlier for health strategies mentioned above in developed countries, the methodology for surveillance of *Healthy China Initiative* has not reached an agreement. Evaluation of health strategies in developed countries generally focus on the enrichment of appraisal dimensions and indicators, as well as the design of quantitative forms to evaluate the performance more comprehensively. However, few attention has been paid to the calculation and comparation of the weights of indicators or the generation of a comprehensive index to synthesize the performance [20]. For example, the strategies of *Healthy Citizen 2020* of US, *Core Health Indicators in the WHO European Region*, *Healthy Japan 2035*, and the relative studies of UK, Australia, and Canada, which all concern about the rich assessment indicators to identify and evaluate their local disease spectrum and health status [4–7, 21–23]. Since it has been only 4 years from the propose of *Healthy China Initiative*, existing research mainly assesses its implementation based on scholars’ interpretation of *Healthy China* with focus on the recognition, value, mechanisms and so on [24–27]. For example, Li and Wang [24] put forward the connotation and the implementation path to promote the construction of *Healthy China* based on the national development status. Pan [28] summarized that most of provinces had carried out corresponding activities and formed effective practice and experience basing on the goals and value of the strategy. With the government emphasis and fast implementation of *Healthy China Initiative*, indicator-based surveillance studies in this field have got increasingly attention. A list of 64 national pilot monitoring indicators has been proposed in 2021, a major challenge in the adoption of this set of indicators resides in that data accessibility and data monitoring capacity have large variations under different region-specific contexts [29], which scholars generally believe that countries or regions to be evaluated have their own advantages and disadvantages in different evaluation items [20]. Domestic research on the methodology of *Healthy China Initiative* surveillance is at the start-up stage while the majority of relevant studies have taken more attention to the theoretical evaluation framework but failed to make full use of the subjective and objective expert consultation, so not to conduct empirical research or there was too much missing data to verify its applicability, like Xiao’s and Wang’s study [30, 31]. Additionally, the indicators involved in the studies were mainly limited to the national formed system and failed to consider the flexibility according to the regions [32–34]. For example, Wang, et al. [35] have established the Healthy China Process Index (HCPI) to evaluate the progress of *Healthy China*, who have not considered the regional characteristics when forming and comparing the index, either did Liu et al. [36]. Regarding research of the empirical application, Wang [35] and Zong [37] have not considered assigning different weights to indicators basing on their importance, which would lead to inaccuracies in the results. And Zhu et al. [38] have failed to produce comprehensive indexes for easy understanding and comparison. In addition, studies mentioned above mainly applied single approach, like literature review or expert consultation or average calculation, to form the indicator system or calculate the index, which would lead to inefficient or inaccuracy in system forming and evaluating. These deficiencies embedded in existing studies all add to the difficulties of investigating the actual performance of *Healthy China Initiative* in different specific regions. However, related studies still provide valuable ideas and methods for us to explore the practical and scientific surveillance approach for *Healthy China Initiative* [39–42].

*Healthy China* is a major national health strategy covering multiple government departments, and an appropriate surveillance process that could be tailored for different region-specific conditions, as well as monitor the progress of the initiative dynamically is essential for achieving nationwide success. Thus, this paper aims to establish a surveillance process including the construction of a multi-dimensional and flexible indicator system and a set of comprehensive indexes for assessing the *Healthy China Initiative*. The surveillance process will be applied in one province of China as a pilot study based on manually collected data from multiple relevant institutions/departments to certify its applicability in revealing the performance of local *Healthy China Initiative* and in providing policy recommendations for policymakers. Moreover, this surveillance process and empirical research would provide inspiration for the future optimization of monitoring and evaluation methods as well as to strengthen *Healthy China Initiative* at different regions.

## Methods

**Fig. 1:**
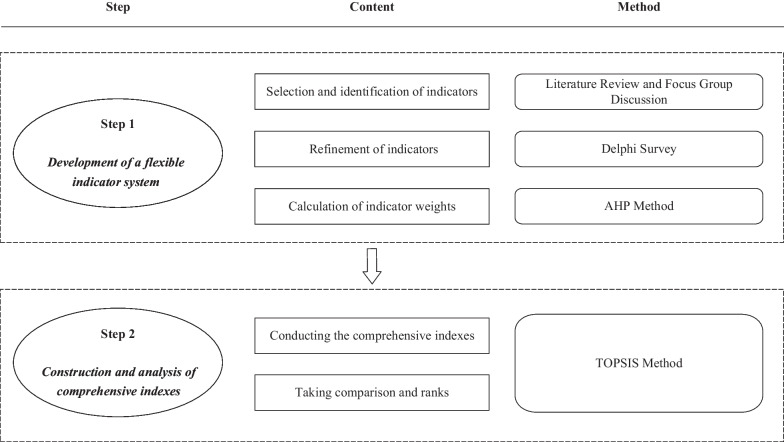
The surveillance process for the *Healthy China Initiative*

The surveillance process included two steps which were (1) the development of a flexible indicator system, and (2) the construction and analysis of comprehensive indexes, as showed in Fig. 1. The surveillance process was then applied in Sichuan Province’s 21 prefecture-level cities/autonomous prefectures as a pilot study.

### Development of a flexible indicator system

The specific indicator system for the target region was established with emphasis on major targets, progress and outcomes of the local *Healthy China Initiative* currently, which could be adjusted according to the diverse conditions of different regions across time. A systemic designing process involving three phases was used to develop the flexible indicator system, which was more time-saving and effective than the simple consultation method [43].

The first phase was the selection and identification of indicators. The establishment of the dimensional frame and the indicator pool was facilitated by literature review referring to searchable existing publications regarding *Healthy China* or relative evaluation from literature platforms, including CNKI, WANFANG Data, PubMed, and Web of Science, relative policies for targeted area from National and local Provincial and Health Commission official websites were also included [13, 17, 44]. The initial indicator list was consisted of major domains of national *Healthy China Initiative* as well as specific indicators available under the context of the target region. Afterwards, 7-rounds of focus group discussions were adopted to determine which indicators should be added into or excluded from the initial indicator list [41, 45]. In total 7 experts from official departments, academe and universities who were familiar with the project very well were invited to join the group discussions and review the indicators based on local realities, and suggestions were also collected from 18 practitioners from provincial and municipal working unit, which was more practical and effective when a novel framework forming. Based on the principles of “representative, well-balanced, accessible”, the peer-reviewed initial indicator list would be confirmed.

The second phase was the refinement of indicators. The Delphi survey, which has been widely adopted in the area of social science and health policy [46–50], was carried out to modify the initial indicator list by enabling experts to propose their opinions independently. Multiple rounds of consultations would be executed iteratively until a consensus was achieved. Experts selected should have enough acquaintance with the *Healthy China Initiative* and specific context of the target region as well as a good knowledge of the indicators and surveillance studies. Also the experts should be selected from multidisciplinary backgrounds to provide diverse sources of opinions [47]. The survey questionnaire would collect experts' basic information as well as self-estimated proficiency in those indicators, and provide a brief introduction to the main contents of the study as well as its relevant evaluation criteria. Eight experts were finally involved in the Delphi survey including policymakers at senior positions from a wide range of health-related institutions and medical schools, and specialists with Ph.D. degrees majoring in health management, health economics, and health policy. The experts involved had 22.5 years of working experience on average. Experts were asked to score each indicator on a 5-point Likert Scale under three main criteria in the two-round survey, namely “Importance”, “Operability” and “Sensitivity” [51, 52], in which a score of ‘‘1’’ indicated not suitable at all and ‘‘5’’ indicated the indicator was totally important, operable, or sensitive. The anonymous feedback was then summarized by combining the experts' positivity coefficient (PC), authority (C), and coordination expressed by the coefficient of variation (CV) and Kendall's coordination coefficient (W) in each round to ensure that all the indicators added to the surveillance indicator system had good performance in terms of representativeness, accessibility, sensitivity, and flexibility [47, 53]. The inclusion criteria were established as follows: PC ≥ 50%, C ≥ 0.70, CV ≤ 0.25, the value of W is around 0.50 or statistically significant, the mean values of “Importance”, “Operability”, “Sensitivity” reached 3.0 [54, 55]. Open-ended questions were set up in each round to gather additional comments from experts. The traditional Delphi method requires 10–50 people and 2 or 3 rounds consultation, but in this study, basing on the combination with multiple rounds of offline group discussions and telephone counseling, taking into full account factors such as time-saving, efficiency and reliability, we made appropriate adjustments regarding the number of online participants in Delphi survey on the basis of reaching consensus and finally reached consistent results after 2 rounds.

The third phase was the calculation of indicator weights. The Analysis Hierarchical Process (AHP), which was formally proposed by Thomas L. Saaty, was adopted for the weights calculation. The method has been proved to be a reliable tool to facilitate systematic and logical decision-making processes, and widely adopted in solving social, industrial and healthy problems via constructing a judgment matrix for analyzing and determining the weights of each criterion [45, 56, 57]. In this research, the 8 experts who had participated in the Delphi method were invited to further rate the weights of indicators in the AHP survey to ensure the effectiveness and the consistency of the consultation as well as the reliability of the results. The main processes included were (1) developing a hierarchical model, (2) constructing pairwise comparison matrixes of indicators, during which experts were required to make judgments on the relative standings of indicators using the Santy 1–9 scale method, (3) calculating the absolute weight of each indicator by Santy's eigenvector procedure and verifying the consistency with a standard of consistency ratio (C.R.) being 0.1 or less, and (4) multiplying the several-dimensions of weights as the combined weights.

### Construction and analysis of comprehensive indexes

TOPSIS (Technique for Order Preference by Similarity to Ideal Solution) is a method for assessing the relative merits of existing objects based on the proximity of a limited number of surveillance objects to an idealized target. It could make full use of information from raw data and the results accurately reflect the gaps between surveillance objects, which could offer the comprehensive indexes as well as indexes under different domains for multi-angle surveillance and is suitable for the situation that the indicators of *Healthy China* are complex while a comprehensive and visualized result is needed. A modified TOPSIS method with weights resulting from the AHP was utilized to construct the comprehensive indexes and facilitate comparison of the outcomes among/within target regions. The main processes were as follow: (1) establishing an original data matrix ($A_{ij}$), where $A_{ij}$ was the original data of the object $j$ for $ith$ indicator, (2) homogenizing the matrix by difference method [40] and normalizing it to a new matrix ($Z_{ij}$), (3) calculating the weighted normalized data matrix ($Q_{ij}$) using indicator weights ($W_{ij}$), as listed in Eq. (1), where $W_{ij}$ was the $ith$ indicator combined weights of the object $j$, (4) determining an optimal solution ($q_{i}^{ + }$) and the worst solution ($q_{i}^{ - }$) of each indicator from the matrix ($Q_{ij}$), the relative proximity of each surveillance object to the optimal and worst solution was used to measure the superiority rank ($D_{j}^{ + }$) and inferiority rank ($D_{j}^{ - }$) of the object $j$, as listed in Eqs. (2), and (5) the comprehensive indexes ($C_{j}$) of each object ranging from 0 to 1 were calculated using Eq. (3), a value closer to 1 indicating a higher rank.

1 $$Q_{ij} = W_{ij} Z_{ij}$$

2 $$D_{j}^{ + } = \sqrt {\sum {(q_{ij} - q_{i}^{ + } )^{2} } }\quad; \, \,D_{j}^{ - } = \sqrt {\sum {(q_{ij} - q_{i}^{ - } )^{2} } }$$

3 $$C_{j} = D_{j}^{ - } /(D_{j}^{ - } + D_{j}^{ + } )$$

Following the proposed surveillance process, publications and documents regarding *Healthy China* and *Healthy Sichuan* from 2015 to 2021 were reviewed, then seven rounds of focus group discussions and two rounds of Delphi survey were conducted. Afterwards, each object could be ranked based on the value of the comprehensive indexes, and the different ranks could be graded. Accordingly, recommendations could be provided to inform the optimization of the initiative-related activities for policymakers.

### Study area and data sources

China is the largest developing country in the world, disparities generally exist in the topography, population distribution, and social-economic development from the northwest to southeast [58]. As the fifth largest province in China in terms of population (83.7 million) and area (486,052 km^2^), Sichuan Province shows similar disparities from the west to east [59], including the landform, uneven distribution of population, per capita GDP and medical resources (see Table 1). Amongst the 21 affiliated regions of Sichuan Province, there are 3 autonomous prefectures where ethnic minorities are dominant and responses differently to national policies among these regions. Therefore, Sichuan Province, which started the *Healthy Sichuan Initiative* in 2019 [44], serves as an ideal area with 21 regions as objects to rehearse the surveillance process as well as help understanding the implementation progress of the initiative considering the variations among regions from 2018 to 2020.

**Table 1:** Basic information of the 21 study regions in Sichuan Province

Region	Resident Population (10,000 person)	Area (km^2^)	GDP Per Capita (CNY)	Urbanization Rate (%)	Number of Medical Institutions	Proportion of Green Areas (%)
Sichuan	8371	486,052	58,126	56.730	82,793	–
Chengdu	2095	14,335	85,679	78.770	11,954	29.030
Zigong	249	4381	58,059	55.400	2162	3.970
Panzhihua	121	7401	85,806	69.570	1009	2.340
Luzhou	426	12,236	50,758	50.240	4727	5.400
Deyang	346	5910	69,443	55.970	2450	2.730
Mianyang	487	20,248	61,936	51.660	4857	4.720
Guangyuan	231	16,311	43,337	47.040	3370	2.720
Suining	282	5323	49,495	57.300	3984	4.340
Neijiang	314	5385	46,228	50.070	3579	2.650
Leshan	316	12,723	63,259	53.110	3224	5.410
Nanchong	561	12,477	42,482	50.220	8248	5.060
Meishan	296	7140	48,132	50.140	2136	2.160
Yibin	459	13,266	61,182	51.390	4993	4.680
Guang'an	326	6341	40,073	44.070	3353	1.930
Dazhou	539	16,582	39,182	49.800	4548	3.920
Ya'an	144	15,046	52,366	52.780	1544	1.760
Bazhong	271	12,293	27,951	46.160	3344	1.830
Ziyang	231	5744	34,806	41.290	3367	1.370
A'ba	82	83,016	49,668	41.430	1728	0.260
Ganzi	111	149,599	36,993	31.010	2813	0.160
Liangshan	486	60,294	35,720	36.960	5403	1.250

The data of 21 study regions was retrieved from Sichuan statistical yearbooks in the end of 2020. “-” means the data is not published

In total, 1848 interagency data covering 3 years from 2018 to 2020 was collected based on the developed indicator system from 8 interagency provincial institutions/departments in the required forms referring to the indicators. The main institutions included were the Health Commission, the Department of Education, the Sports Bureau, the Department of Civil Affairs, the Department of Ecology and Environment, the Department of Water Resources, the Department of Agriculture and Rural Affairs, and the Administration of Traditional Chinese Medicine in Sichuan. Among that, the data of 16 indicators was collected from different divisions of the Health Commission, including the Maternal and Child Health Division, the Disease Prevention and Control Division, the Major Infectious Disease Prevention and Control Division, the Occupational Health Division, the Elderly Health Division, the Primary Health Care Division, the Planning and Finance Division, the Publicity and Promotion Division, and the Patriotic Health Campaign Committee Office. And the rest data was obtained from other 7 provincial departments as listed above.

## Results

### The indicator system for *Healthy China Initiative* in Sichuan Province

The response rates of the eight experts in the two-round Delphi survey following the focus group discussions and phone-consultation were as high as 100% and 87.5%, indicating that the experts had high motivations to support the study. The expert's authority coefficients were found to be 0.919 and 0.900 respectively, proving that the experts were familiar enough with the research and the scores were reliable. The mean scores and CV of “Importance” “Operability” and “Sensitivity” all met the inclusion criteria, and Kendall's coordination coefficients W were statistically significant, indicating that the indicators were reasonably set up and reached an agreement between the experts. According to the open-ended feedback, the expression and calculation of two indicators were amended, and the titles of three domains were revised. The weights of the final indicators calculated through AHP all passed the consistency test (C.R. < 0.1).

Finally a current specific indicator system for *Healthy China Initiative* in Sichuan with 5 primary domains and 23 secondary indicators and corresponding weights was constructed (see Table 2). Meanwhile, 14 *Healthy Sichuan Special Campaigns* responding to the national special campaigns were included in the indicator system. Specifically, the “Health Level” domain was related to the Special Campaign “Promotion of Maternal and Child Health” and reflected the improvement of people's health level throughout their life cycle. The “Healthy Living” domain was related to the Special Campaigns “Healthy Knowledge Popularization” “Health Promotion in Schools” “National Fitness” and reflected the effective control of major health risk factors and the improvement of health literacy and lifestyles. The “Disease Prevention and Control” domain involved nine Special Campaigns of “Prevention and Control of Infectious diseases and Endemic diseases” “Occupational Health Protection” “Health Promotion of the Elderly” “Prevention and Control of Cardiovascular Diseases/Cancer/Chronic Respiratory Diseases/Diabetes” “Mental Health Promotion” “Promotion of Health in the Chinese Treatment of Premature Diseases”, these campaigns aimed at improving the health of critical populations and the physique of the entire population through establishing a comprehensive basic healthcare system [24]. Specifically, for the 15th indicator “Development of Traditional Chinese Medicine”, we finally set three sub-indicators under it, which could embody the concept of “Traditional Chinese Medicine” and also help understand the specific content and take measurement of the “Development of Traditional Chinese Medicine” more clearly and intuitively basing on the national and provincial assessment indicators, the experts’ opinions, and data availability. The “Health Service” domain reflected the development of healthcare delivery system, security system, and governance capacity in the target region, all of them aimed at building a diversified and sustainable healthcare system to ensure access to effective and equitable health services for the public [60]. The “Healthy Environment” domain was related to the Special Campaign “Promoting Healthy Environment” and reflected the upturn of health-friendly production and living environment [24].

**Table 2:** Indicator system and data information for *Healthy China Initiative* in Sichuan Province

Domain	Weight of domain	No	Symbol	Indicator	Connotation	Weight of indicator	combined weight	Data source (Division)	Data form
Health level	0.384	1	*#	Infant mortality (‰)	The proportion of infant deaths in a region	0.438	0.168	Health Commission (MCH)	Department reporting data
		2	*#	Maternal mortality (per 100,000 population)	The proportion of maternal deaths in a region	0.339	0.130	Health Commission (MCH)	Department reporting data
		3	*#	Life expectancy per capita (years)	The average number of years a person is expected to survive at birth	0.222	0.085	Health Commission (DPCD)	Department reporting data
Healthy living	0.188	4	*#	Health literacy level (%)	The proportion of people with health literacy in the total population	0.548	0.103	Health Commission (PPD)	Department reporting data
		5	*	Qualified rate of National Student Physical Health Standard (%)	The proportion of students meeting the excellent standard participating in the evaluation	0.195	0.037	Department of Education	Department reporting data
		6	*#	Percentage of residents who have reached the National Physical Fitness Standard (%)	Percentage of residents in the province who have achieved the passing grade of National Physical Fitness Standard and above	0.257	0.048	Sports Bureau	Department reporting data
Disease prevention and control	0.157	7	*#	Immunization program vaccination rate for school-age children by township (%)	Vaccination rate of school-age children within the immunization plan in the township(town, street)	0.273	0.043	Health Commission (DPCD)	Department reporting data
		8		Incidence of tuberculosis (per 100,000 population)	The proportion of new cases of tuberculosis reported in a certain area and population over some time(usually 1 year)	0.112	0.018	Health Commission (MIDPCD)	Department reporting data
		9	*#	The rate of new pneumoconiosis cases in workers with exposure to dust less than 5 years (%)	The proportion of newly reported cases of pneumoconiosis among workers with less than 5 years of dust exposure	0.069	0.011	Health Commission (OHD)	Department reporting data
		10		Health management rate of the elderly over 65 years old (%)	The proportion of permanent residents aged 65 and above who received standardized health management	0.127	0.020	Health Commission (EHD)	Department reporting data
		11	*#	Premature mortality of cardiovascular disease, cancer, chronic respiratory disease, and diabetes in people aged 30–70 (%)	The proportion of deaths from the cardiovascular disease, cancer, chronic respiratory disease, and diabetes in people aged 30–70 years old	0.146	0.023	Health Commission (DPCD)	Department reporting data
		12	#	Early diagnosis rate of critical cancer species in high prevalence areas (%)	Percentage of early diagnosis of critical cancers among people living in high incidence areas	0.099	0.015	Health Commission (DPCD)	Department reporting data
		13		The avoidable hospitalization rates for Ambulatory Care Sensitivity Conditions (selected as Essential Hypertension and Type 2 Diabetes)(per 100,000 population)	The hospitalization proportion of ambulatory care sensitivity disease among the sick population in a given area	0.064	0.010	Health Commission (PHCD)	Calculating from individual data
		14		The proportion of counties with community rehabilitation services for mental disorders (%)	The proportion of counties that have established community rehabilitation services for mental disorders	0.057	0.009	Department of Civil Affairs	Department reporting data
		15	*#	Development of Traditional Chinese Medicine	The proportion of TCM hospitals with departments for preventive treatment of disease(%)	0.054	0.008	Administration of Traditional Chinese Medicine	Department reporting data
					The proportion of township health centers and community health service centers(or village health offices) providing 6 (or 4) TCM non-pharmacological treatment services(%)	0.054	0.008		
					The coverage rate of TCM health management services for children aged 0–36 months or elderly aged 65 or above(%)	0.054	0.008		
Health Service	0.145	16		Amount of total government health expenditure (CNY)	The government's expenditure for various types of medical and health service	0.451	0.065	Health Commission (PFD)	Department reporting data
		17	*#	Personal health expenditure accounting for the proportion of total health expenditure (%)	The proportion of the personal burden in receiving various types of medical and health service	0.228	0.033	Health Commission (PFD)	Department reporting data
		18	*#	Numbers of psychiatric practitioners (assistant) (per 1000 population)	Numbers of practicing (assistant) physicians per 1000 resident population	0.194	0.028	Health Commission (PFD)	Department reporting data
		19	#	Numbers of registered nurses(per 1,000 population)	Numbers of registered nurses per 1,000 resident population	0.127	0.018	Health Commission (PFD)	Department reporting data
Healthy Environment	0.127	20	#	The proportion of days with a good and excellent air quality (%)	The proportion of days with an air pollution index at or above the National Quality Secondary Standard	0.397	0.050	Department of Ecology and Environment	Department reporting data
		21		The coverage rate of Hygienic City and Hygienic Township(%)	The proportion of Hygienic City and Hygienic Township among the cities or townships in the area	0.195	0.025	Health Commission (PHCCO)	Department reporting data
		22	#	Penetration rate of piped water in rural(%)	The proportion of the rural population(including neighborhoods or yards) covered by rural or urban centralized water supply and extension projects	0.228	0.029	Department of Water Resources	Department reporting data
		23	#	Penetration rate of the sanitary toilets in rural(%)	The proportion of rural households using sanitary toilets among total toilets which should be built	0.180	0.023	Department of Agriculture and Rural Affairs	Department reporting data

The indicators marked “*” in “Symbol” have been included in the provincial government assessment indicators, the indicators marked “#” are listed in the *Healthy China Initiative* 64 indicators. In the “Data source (Division)”, Maternal and Child Health Division is abbreviated as “MCHD”, Planning and Finance Division is abbreviated as “PFD”, Disease Prevention and Control Division is abbreviated as “DPCD”, Elderly Health Division is abbreviated as “EHD”, Publicity and Promotion Division is abbreviated as “PPD”, Primary Health Care Division is abbreviated as “PHCD”, Occupational Health Division is abbreviated as “OHD”, Major Infectious Disease Prevention and Control Division is abbreviated as “MIDPCD”, Patriotic Health Campaign Committee Office is abbreviated as “PHCCO”

As shown in Table 2, the “Health Level” domain had the greatest influence among the five domains with a weight of 0.384, the rest domains had a more balanced influence with the weights being 0.188, 0.157, 0.145 and 0.127 respectively. Amongst the secondary indicators, the four with greatest weights were “infant mortality” “maternal mortality” “health literacy level” and “life expectancy per capita”, indicating that these indicators had greater influences on *Healthy Sichuan Initiative*. Meanwhile, “development of Traditional Chinese Medicine” “the proportion of counties with community rehabilitation services for mental disorders” and “the avoidable hospitalization rates for Ambulatory Care Sensitivity Conditions (selected as Essential Hypertension and Type 2 diabetes)” [61, 62] were the 3 indicators with the lowest weights, reflecting that these indicators either had less impact on *Healthy Sichuan Initiative* or their concepts/influence had not been popularized and attracted enough attention. Among that, experts just weighted the indicator “Development of Traditional Chinese Medicine” to represent the relative importance of the TCM including the three sub-specific indicators in the AHP survey without weighting the sub-three indicators separately and they reached a consensus.

### Analysis of *Healthy China Initiative* in Sichuan Province

In total, 1848 interagency data covering 3 years from 2018 to 2020 was collected based on the developed indicator system from 8 provincial institutions/departments in the required forms referring to the indicators (see Table 2). Among that, data for 16 indicators was collected from different divisions of the Health Commission. The rest data was obtained from other 7 provincial departments as listed in Table 2. Most of the data collected was the department reporting data which could be used directly for analyzing, except the data for the indicator “the avoidable hospitalization rates for Ambulatory Care Sensitivity Conditions (selected as Essential Hypertension and Type 2 Diabetes)”. Value for this indicator was calculated using individual inpatient discharge data and represented as the proportion of admissions in total diagnosed patients with Essential Hypertension or Type 2 Diabetes, with inspiration from Zhao and Chen [61, 62].

The comprehensive indexes for the 21 affiliated regions were calculated yearly by TOPSIS, which were further ranked, as shown in Table 3. In general, the comprehensive indexes showed disparities between regions with updating in Sichuan Province, while the gaps among regions shrank slowly after 2019. At the same time four out of the five domains showed clear upward turning points except for the “Healthy Environment” domain. Specially, under the “Health Level” domain, the three autonomous prefectures (ethnic minority areas) which were impoverished and remote had the lowest indexes across three years. The fluctuations were relatively small and uniform across years for the “Healthy Living” and “Healthy Environment” domains which relating to the living habits and natural conditions. Inflection points with data appeared in all regions by the end of 2019 under the “Disease Prevention and Control” and “Health Service” domains which were vulnerable to social factors. The gaps of the comprehensive indexes ranged from 0.773 to 1.000 among regions under each domain and from 0.349 to 0.941 between different domains. Besides, “Early diagnosis rate of critical cancer species in high prevalence areas” was involved in 9 prefecture-level cities which were analyzed separately, among that only 5 were able to provide complete datasets over the study period, whose indexes showed upward trend for 4 regions.

**Table 3:** Surveillance outcomes of *Healthy China Initiative* in 21 regions

Region	Totally		Health Level		Healthy Living		Disease Prevention and Control		Health Service		Healthy Environment	
	Index	Rank	Index	Rank	Index	Rank	Index	Rank	Index	Rank	Index	Rank
*In 2018*												
Chengdu	0.762	1	1.000	1	0.993	1	0.584	2	0.376	8	0.936	1
Zigong	0.178	18	0.940	4	0.161	19	0.404	9	0.107	21	0.178	13
Panzhihua	0.292	8	0.788	16	0.745	2	0.360	13	0.682	2	0.156	16
Luzhou	0.355	5	0.909	8	0.498	8	0.407	8	0.664	4	0.254	6
Deyang	0.204	15	0.909	7	0.406	11	0.374	12	0.212	16	0.183	12
Mianyang	0.287	9	0.970	2	0.364	12	0.579	3	0.348	10	0.249	7
Guangyuan	0.304	7	0.940	3	0.083	20	0.403	10	0.677	3	0.175	14
Suining	0.198	16	0.909	6	0.238	16	0.280	17	0.260	11	0.172	15
Neijiang	0.193	17	0.849	11	0.268	15	0.301	15	0.133	18	0.197	10
Leshan	0.210	13	0.758	18	0.425	10	0.261	18	0.212	15	0.198	8
Nanchong	0.326	6	0.909	5	0.344	13	0.351	14	0.216	14	0.347	4
Meishan	0.216	12	0.879	9	0.314	14	0.908	1	0.189	17	0.151	17
Yibin	0.411	2	0.819	14	0.523	7	0.438	7	0.814	1	0.268	5
Guang'an	0.260	11	0.819	13	0.001	21	0.526	4	0.420	5	0.197	9
Dazhou	0.377	3	0.849	10	0.175	17	0.453	6	0.118	20	0.434	2
Ya'an	0.206	14	0.758	17	0.597	6	0.292	16	0.364	9	0.129	19
Bazhong	0.267	10	0.819	12	0.479	9	0.454	5	0.399	6	0.185	11
Ziyang	0.155	21	0.788	15	0.173	18	0.403	11	0.125	19	0.143	18
A'ba	0.166	20	0.029	21	0.657	5	0.165	19	0.255	12	0.095	21
Ganzi	0.175	19	0.483	19	0.693	3	0.095	21	0.241	13	0.117	20
Liangshan	0.373	4	0.214	20	0.666	4	0.111	20	0.396	7	0.363	3
*In 2019*												
Chengdu	0.859	1	0.947	3	0.993	1	0.561	3	0.693	5	0.935	1
Zigong	0.210	18	0.905	7	0.071	20	0.245	18	0.346	21	0.170	14
Panzhihua	0.279	11	0.723	15	0.663	3	0.350	14	0.727	3	0.150	17
Luzhou	0.354	5	0.866	11	0.471	10	0.411	10	0.697	4	0.265	6
Deyang	0.239	17	0.854	12	0.513	6	0.507	5	0.370	19	0.175	12
Mianyang	0.309	8	1.000	1	0.670	2	0.559	4	0.545	10	0.231	7
Guangyuan	0.280	10	0.914	6	0.110	19	0.423	7	0.682	6	0.168	15
Suining	0.251	14	0.868	10	0.165	15	0.273	17	0.585	8	0.173	13
Neijiang	0.251	15	0.813	14	0.159	17	0.339	15	0.453	12	0.191	9
Leshan	0.261	13	0.602	18	0.369	12	0.283	16	0.428	13	0.214	8
Nanchong	0.329	6	0.871	9	0.170	14	0.378	13	0.424	14	0.310	4
Meishan	0.244	16	0.917	5	0.298	13	0.915	1	0.362	20	0.163	16
Yibin	0.396	4	0.903	8	0.451	11	0.415	8	0.819	2	0.296	5
Guang'an	0.283	9	0.943	4	0.002	21	0.622	2	0.636	7	0.182	10
Dazhou	0.427	2	0.713	16	0.161	16	0.404	12	0.416	15	0.438	2
Ya'an	0.267	12	0.665	17	0.544	5	0.469	6	0.566	9	0.127	19
Bazhong	0.326	7	0.852	13	0.491	8	0.408	11	0.834	1	0.178	11
Ziyang	0.200	20	0.964	2	0.117	18	0.411	9	0.410	16	0.130	18
A'ba	0.206	19	0.062	21	0.579	4	0.115	19	0.383	18	0.126	20
Ganzi	0.194	21	0.508	19	0.510	7	0.113	20	0.395	17	0.103	21
Liangshan	0.400	3	0.233	20	0.481	9	0.093	21	0.474	11	0.388	3
*In 2020*												
Chengdu	0.910	1	0.907	9	1.000	1	0.609	4	0.798	4	0.951	1
Zigong	0.193	17	0.785	16	0.066	19	0.486	9	0.412	17	0.142	12
Panzhihua	0.214	12	0.800	15	0.666	3	0.451	11	0.640	5	0.124	17
Luzhou	0.256	8	0.850	13	0.495	9	0.423	14	0.615	10	0.193	7
Deyang	0.186	19	0.851	12	0.512	7	0.559	7	0.202	21	0.154	9
Mianyang	0.256	7	0.967	5	0.780	2	0.576	6	0.458	16	0.199	6
Guangyuan	0.206	14	0.958	6	0.050	20	0.432	13	0.633	6	0.132	14
Suining	0.200	16	0.988	3	0.119	18	0.486	10	0.524	13	0.150	10
Neijiang	0.203	15	0.929	7	0.156	16	0.377	18	0.624	8	0.130	15
Leshan	0.217	11	0.766	17	0.358	12	0.270	20	0.629	7	0.148	11
Nanchong	0.277	5	0.997	1	0.178	14	0.398	15	0.349	18	0.269	4
Meishan	0.212	13	0.989	2	0.212	13	0.832	1	0.502	14	0.136	13
Yibin	0.317	3	0.891	10	0.450	11	0.579	5	0.819	3	0.228	5
Guang'an	0.219	10	0.926	8	0.011	21	0.614	3	0.609	11	0.155	8
Dazhou	0.330	2	0.886	11	0.161	15	0.487	8	0.270	20	0.335	2
Ya'an	0.242	9	0.729	18	0.545	5	0.382	16	0.956	1	0.104	18
Bazhong	0.266	6	0.847	14	0.520	6	0.710	2	0.915	2	0.130	16
Ziyang	0.190	18	0.985	4	0.127	17	0.436	12	0.618	9	0.103	19
A'ba	0.137	21	0.000	21	0.574	4	0.349	19	0.273	19	0.075	20
Ganzi	0.181	20	0.412	19	0.467	10	0.382	17	0.541	12	0.067	21
Liangshan	0.299	4	0.334	20	0.496	8	0.147	21	0.488	15	0.269	3

Regarding the relative ranks of the regions, Chengdu which is the provincial capital city ranked No.1 for consecutive three years and could be recognized as the pioneer in the *Healthy Sichuan Initiative*. Besides that, several regions showed uprising turns of ranks by the end of 2019, while the others showed an opposite change pattern. The ranks showed significant fluctuations under four out of the five domains except for the “Healthy Environment” domain whose rank was relatively stable.

We also applied the Fuzzy Comprehensive Evaluation (FCE) method as a sensitivity analysis, which was simpler in calculation to get comprehensive outcomes [63]. The outcomes were partly shown in Table 4. The results of the FCE and TOPSIS method generally showed the same trend with some differences in ranks of several regions, which might be related to the sensitivity of different methods. Finally, we utilized TOPSIS method in this study considering its objectivity, truthfulness, and reliability referring to existing literatures [40, 64].

**Table 4:** Surveillance outcomes of *Healthy China Initiative* in the 21 regions with FCE method

Time	2018		2019		2020	
Region	Total Score	Rank	Total Score	Rank	Total Score	Rank
Chengdu	69.553	1	73.366	1	80.336	1
Zigong	56.698	20	57.561	20	62.058	19
Panzhihua	62.419	5	62.901	9	63.892	11
Luzhou	63.667	3	64.469	4	65.252	5
Deyang	59.255	13	60.766	16	61.806	20
Mianyang	61.273	8	63.406	6	65.139	6
Guangyuan	62.508	4	63.094	7	63.845	12
Suining	58.512	15	60.791	15	62.989	17
Neijiang	57.729	17	60.487	17	63.316	16
Leshan	58.332	16	60.889	14	63.716	13
Nanchong	59.897	12	61.514	13	63.425	14
Meishan	59.974	11	61.561	12	65.079	7
Yibin	63.844	23	65.779	2	67.638	2
Guang'an	60.276	10	62.504	10	64.034	10
Dazhou	60.835	9	63.075	8	64.740	8
Ya'an	58.883	14	61.781	11	65.252	4
Bazhong	61.693	6	65.078	3	67.110	3
Ziyang	57.292	18	59.701	18	63.368	15
A'ba	56.596	21	57.556	21	59.010	21
Ganzi	56.995	19	58.027	19	62.154	18
Liangshan	61.665	7	63.560	5	64.405	9

FCE is short for the Fuzzy Comprehensive Evaluation

## Discussion

### Applicability of the proposed surveillance process

A well-designed indicator-based surveillance process serving as a *Healthy China Initiative* tracker has been proven an effective instrument with the rationality of the indicator system and the application of evaluation results via literature comparisons and the pilot study to comprehensively report the performance of the initiatives and guide new interventions [65].

A combination of literature review, multiple group discussions and Delphi consultation was used to propose the indicator system, which integrated online-consultation with face-to-face interview and the balance of subjective and objective opinions, and the comprehensive indexes were calculated more accurately by AHP and TOPSIS method. Compared with the existing methods, the two-step surveillance process could monitor the progress more practical and cost-effective, and is more focused with considering the flexibility for different area. For example, Wang et al. [31] have constructed the evaluation indicator system for *Healthy China* with 170 specific indicators in 6 dimensions based on literature review, but only 91 indicators had interrupted data collected, and comprehensive measurement could not be carried out. Wang et al. [35] have used Delphi method, multilevel averaging weights and geometric method to establish the indicator system and account for the HCPI, while the final system just contained 13 indicators in 5 domains and were set unevenly, either the average weights were used without considering the relative importance of different indicators causing the misleading results. In addition, Chen et al. [66] analyzed the absolute values of 15 indicators of the *Healthy Guangxi Initiative* in 2020 without comprehensive measurement, which lacked systemic and accuracy. And Zeng et al. [67] just analyzed the data related to the “Health Level” domain of Jiangxi Province. Our study takes into account the surveillance process of comprehensiveness, operability, flexibility, reliability and efficiency which would lead to more accuracies in evaluation compared with the previous studies [35–37].

The pilot study carried out in Sichuan Province verified the applicability and superiority of the proposed progress. A specific indicator system for *Healthy China Initiative* in Sichuan was first established and was applicable in analysis, which covered important indicators listed in above studies as well as adjusted specific indicators according to the local. The surveillance outcomes could be used to provide recommendations for further schemes and modification of activities at the target regions with in-depth understanding of their bottlenecks and barriers under the region-specific contexts, also joint efforts between departments can be facilitated in an effective mode [46, 68]. The evidence from Sichuan Province showed that the penetration of the *Healthy China Initiativ*e had a significant impact in Sichuan Province with the inflection points of surveillance outcomes at the end of 2019 when the initiative started, but more efforts are still needed given the 3-years unsatisfactory outcomes and the relatively slow implementation status. As indicated by the indexes, large disparities displayed among different regions in Sichuan Province, especially between the developed and less developed regions [69, 70], which were likely to be affected by various factors including the capacity of the local government, socioeconomic factors, geographical environment, and the impact of COVID-19 [71]. Furthermore, we found that inflection points appeared in all regions by the end of 2019 to 2020 based on the evaluation results, especially in the “Disease Prevention and Control” and “Health Service” domains. We conducted a preliminary analysis of the results and the relative raw data, and found that in the domain of “Disease Prevention and Control”, all regions showed upward trends with varying degrees from 2019 to 2020 except one city. For the “Health Service” domain, most of the regions had improved their health service level except for three regions from 2019 to 2020. We speculated that the governments might have strengthened their disease control relative works and have taken different measures in response to the epidemic, and have taken more attention to the public health and improved support for medical and healthcare from 2019 to 2020 thus resulting in the data changes at the time.

### Implication for promotion of *Healthy China Initiative*

An effective surveillance process is useful to facilitate the dynamic monitoring of health-related data as well as real-time reporting, and to encourage the improvement and cooperation from intersectoral activities, which is very important to promote the *Healthy China Initiative* and help the achievement of *Healthy China* strategy.

The deficiency was clear that there was still no sound data collection system and monitoring process when we took the surveillance. We conducted the data gathering procedure through issuing governmental documents and retrieving data from multiple departments, which proved to be time-consuming and inefficient. Moreover, due to the lack of a well data monitoring system to mandate the reporting of data regularly, the problem of data missing and poor data quality generally exists. That is also the reason why the current national pilot indicators cannot be widely applied, which is the obstacle for grasping the process. Statistical information platforms or big data technologies, also a well-structured data collection system and processing techniques are desperately needed to improve the reliability and quality of surveillance data, which could be useful to draw attention to the activities easily overlooked. Based on the progress of *Healthy China Initiative* and the changes of priority tasks, the indicator system could be adjusted and improved accordingly to take supervision more efficiently following the process.

Results from the surveillance process should be utilized to guide the implementation and propose the *Healthy China Initiative*. Evidence of surveillance outcomes of Sichuan Province revealed that delays and gaps generally existed in the implementation of *Healthy China Initiative* related policies/activities, which added to the disparities of regional progress towards *Healthy China*. Under such circumstances, self-examination and self-rectification based on the surveillance outcomes are essential for the confirming of major problems and weaknesses in target regions, thus push forward the *Healthy China Initiative* and narrow the gaps among regions. Review of the surveillance outcomes should be connected to local rewarding and punishing mechanisms. And the surveillance process should also be implemented to explore the potential innovative activities instead of merely relying on governmental interventions from above.

As the upmost national health strategy, *Healthy China* requires *Integration of health into all policies*, which cannot be achieved without the joint efforts of departments and divisions at the governmental, societal, and individual levels. The surveillance process would facilitate the collaboration among different departments for the implementation of *Healthy China Initiative*. For instance, government plays a key role in guiding the implementation of initiative-related activities in terms of identifying problems according to surveillance outcomes, providing problem-targeted interventions promptly, and making predictions of the dynamic evolvement of the initiatives from policymakers’ perspective, also the investment-related decisions, and valuable empirical experience from other provinces or prefectures summarized and learnt [72–74]. Individuals would like to strengthen exercises and rehabilitations to reduce the incidence of chronic non-communicable diseases as well as improve health literacy [7]. The healthcare institutions should provide high-quality and efficient health services. Furthermore, the social media platforms should be widely adopted to facilitate the interpretation and propaganda of *Healthy China Initiative* to raise public awareness of health-related issues.

### Study limitations

Several limitations need to be noted in this practice. First, the indicators added to the system are not yet complete to include all possible relative indicators mainly due to data limitation, such as “dental caries rate in children”. Second, there exist some missing data due to the lack of a well-structured data monitoring system which was not included in the analysis, and some indicators were just proposed with no data, such as “the proportion of TCM hospitals with departments for preventive treatment of disease”. Third, the data collection approach manually might have caused the data deviation during the transferring procedures. Forth, an improved-Delphi method by combining focus group discussions and telephone consultation with the Delphi email survey was used to ensure the reliability and efficiency of the results, and the final number of online experts included in this survey was constrained to 8 based on expert interviews, which has not met the requirement in traditional Delphi method which needs 10–50 people. Fifth, because of the shortage of data, there was a lack of more tangible evidence to confirm that the epidemic did have an impact and we did not specifically analyze the mechanism of how the 2020 data were affected by different factors including the epidemic in this study, and perhaps further studies would be explored in the future studies.

## Conclusions

A surveillance process consisting of the development of a flexible indicator system and construction of a set of comprehensive indexes was developed for monitoring and evaluation of the *Healthy China Initiative*. Apart from monitoring and assessing the performance of current *Healthy China Initiative* at different regions, this process also provides a theoretical foundation as well as empirical evidence to facilitate the sustainability of surveillance-related activities to support the penetration of *Healthy China Initiative* and achieve the agenda of *Building and Sharing Health for All* across the nation from a long-term perspective. The proposed surveillance process would bring us one step closer to better evaluation frameworks and methods with improved effectiveness and reliability at different regions. The in-depth empirical analysis involving large amount and multi-dimension data relevant to the implementation of *Healthy China Initiative* from diverse government departments as well as the series of policy implications provided in the study has great potential to inform the penetration of *Healthy China Initiative* in other provinces.

## Data Availability

Basic information of 21 study regions was retrieved from Sichuan statistical yearbooks of 2020 obtained from Sichuan Provincial Bureau of Statistics. The data utilized for assessing the implementation of *Healthy China Initiative* was collected from multiple provincial institutions/departments of Sichuan by permission for the current study, which cannot be shared publicly.
